# Accurate electromechanical characterization of soft molecular monolayers using piezo force microscopy†

**DOI:** 10.1039/c9na00638a

**Published:** 2019-11-01

**Authors:** Nathaniel C. Miller, Haley M. Grimm, W. Seth Horne, Geoffrey R. Hutchison

**Affiliations:** Department of Chemistry, University of Pittsburgh Pennsylvania 15260 USA geoffh@pitt.edu

## Abstract

We report a new methodology for the electromechanical characterization of organic monolayers based on the implementation of dual AC resonance tracking piezo force microscopy (DART-PFM) combined with a sweep of an applied DC field under a fixed AC field. This experimental design allows calibration of the electrostatic component of the tip response and enables the use of low spring constant levers in the measurement. Moreover, the technique is shown to determine both positive and negative piezo response. The successful decoupling of the electrostatic component from the mechanical response will enable more quantitative electromechanical characterization of molecular and biomaterials and should generate new design principles for soft bio-compatible piezoactive materials. To highlight the applicability, our new methodology was used to successfully characterize the piezoelectric coefficient (*d*_33_) of a variety of piezoactive materials, including self-assembled monolayers made of small molecules (dodecane thiol, mercaptoundecanoic acid) or macromolecules (peptides, peptoids), as well as a variety of inorganic materials, including lead zirconate titanate [PZT], quartz, and periodically poled lithium niobate [PPLN]. Due to high differential capacitance, the soft organic monolayers demonstrated exceedingly large electromechanical response (as high as 250 pm V^−1^) but smaller *d*_33_ piezocoefficients. Finally, we find that the capacitive electrostatic response of the organic monolayers studied are significantly larger than conventional inorganic piezoelectric materials (*e.g.*, PZT, PPLN, quartz), suggesting organic electromechanical materials applications can successfully draw from both piezo and electrostatic responses.

## Abstract 2

We report a new methodology for the electromechanical characterization of organic monolayers based on the implementation of dual AC resonance tracking piezo force microscopy (DART-PFM) combined with a sweep of an applied DC field under a fixed AC field.

## Graphical abstract

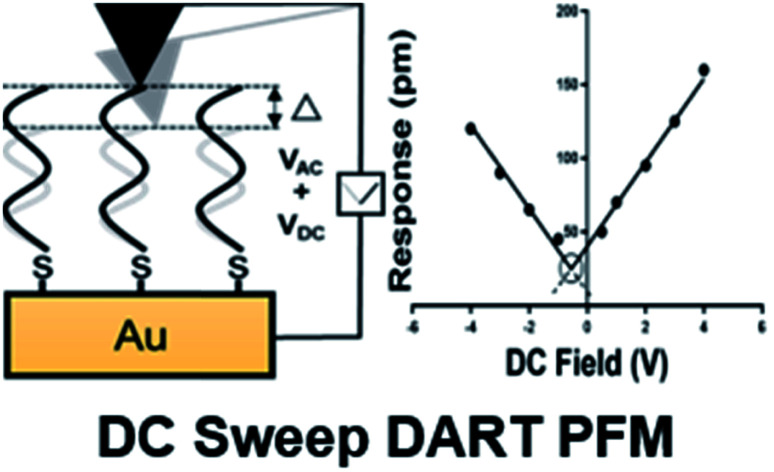

Since the discovery of piezoelectric activity in muscle tissue and other biological materials, the molecular origin of the electromechanical response has been a topic of interest. At the nanoscale, the electrical and mechanical properties of materials are often linked – for example giving rise to phenomena such as piezo-, flexo-, and ferroelectricity.^1–5^ These phenomena, in turn, enable a wide range of applications from sensing to optoelectronics.^3,4,6–15^ The piezoelectric effect (PE) comprises two effects: a direct effect, in which mechanical stress generates an electric charge. Inversely, the converse PE generates a mechanical response to an applied electric field. Materials exhibiting piezoelectric response are generally non-centrosymmetric, polar, and poorly conductive. A range of materials exhibit piezoelectric properties including lead zirconate titanate (PZT), quartz, and various polymers such as polyvinylidene difluoride (PVDF). At the nanoscale the lack of centrosymmetry coupled with high polarities give rise to piezoelectric response, yielding a vast diversity of piezoelectric materials. For example, self-assembled monolayers, where the attachment of target molecules to surfaces inherently breaks symmetry and generates a polar system, have been shown recently to be inherently piezoelectric.^16^

Accurate and reliable methods to measure piezoelectric outputs from a given material are vital to investigating these phenomena and realizing their potential range of applications. Atomic force microscopy (AFM) was initially developed to map the morphological variations in materials at the nanoscale.^6,17^ Beyond simple topology and morphology, functional AFM methods have been developed to map properties including surface potential, charge transport, magnetic response, and piezoresponse.^17–21^ The latter, piezo force microscopy (PFM), determines the mechanical response of materials to an applied electrical field by measuring the converse piezoelectric effect. However, classical single frequency PFM suffers from low sensitivity and poor frequency tracking due to crosstalk in the phase feedback loop between material topology and electromechanical response. To increase sensitivity and avoid dielectric breakdown of materials, dual AC resonance tracking (DART) was developed by Kalinin to allow the use of small bias voltages while maintaining good frequency tracking despite varying topological features.^4,6,18,20–24^ Building on the principles of PFM, DART drastically improved the sensitivity of PFM measurements and helped move the field towards more quantitative piezoelectric measurements. Beyond DART, the band excitation (BE) method was intended to overcome distortions associated with tip–sample interactions experienced in DART, in which the lever is excited at multiple frequencies around the fundamental frequency to alleviate shifts in the fundamental, due to topography.^25^ More recently, several groups have tried to reduce/eliminate these distortions by using high spring-constant (*k*_l_) levers, with or without a fixed external DC field, or by creating new lever technologies, such as “inner paddled levers”.^2,3,26–28^ These techniques reduce the electrostatic component of the measurement for specific cases; however, this may not be true for systems, such as organic polymers and biomaterials, in which the electrostatic component is quite large or where the Young's modulus of the material is small in comparison to the lever.

The above methods, particularly the use of high spring-constant levers, perform best with materials in which the elastic modulus is significantly higher than that of the lever. Unfortunately, when the modulus of the material under study is small in comparison to modulus of the lever, such as organic and biomaterials, the lever may deform the target surface, reducing or eliminating the sensitivity enhancements garnered by DART or band excitation techniques.

In this work, we describe a method for improved accuracy in measurements of the piezo-response (*d*_33_, the response of a material in the *z*-axis to a field applied in the same axis) of soft organic monolayers. The method entails the use of a soft (low-*k*_l_) lever, coupled with the quantification of the electrostatic component of tip response by completing a DC field sweep in addition to the AC field sweep already employed to measure the independent lever electrostatics. By compensating for the electrostatic component, the true *d*_33_ of the material can be established.

## Results and discussion

**Fig. 1:**
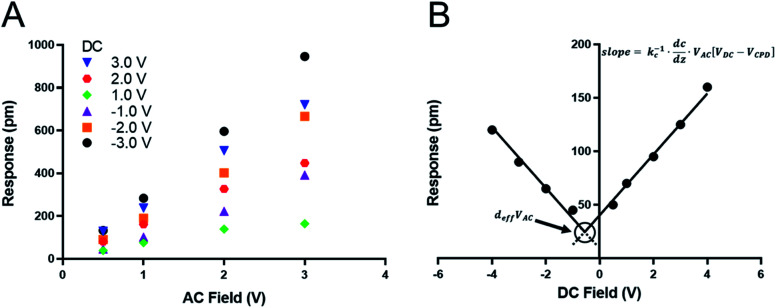
(A) Traditional determination of piezoresponse using piezo-force microscopy by varied *V*_AC_ – the slope of the trend line should reflect the *d*_eff_ piezoresponse (pm V^−1^). (B) Suggested *V*_DC_ sweep technique to determine the piezo response in soft-organic piezo materials – the crossing point reflects the *d*_eff_ piezoresponse, and the slope reflects the electrostatic contribution of the material.

We recently measured the piezoresponse of fixed polar molecular self-assembled monolayers, anchored by gold–thiol interactions to gold-coated glass substrates.^16^ These well-formed monolayers represent model systems for the investigation and development of soft, flexible, fixed polar organic piezoelectric materials.^29^ In that work, piezoresponse was determined using DART-PFM by sweeping the applied AC electric field and plotting the corresponding measured response against it. The slope of the linear regression should yield the effective *d*_33_ (*d*_eff_), in recognition of the lack of direct measurement of the true fields experienced by the material and minor yet contributing electromechanical effects, of the material under study, as illustrated in Fig. 1A. Unfortunately, the regression rarely passes through the origin due to electrostatic effects present when the tip is brought into contact with the surface; resulting in a sizeable inherent error in the measurement regardless of the care taken in the data acquisition.

The tip response can be determined as in eqn (1)^17,24^1
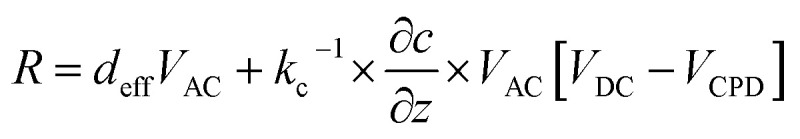
where cantilever response *R* is equal to the *d*_eff_ (effective piezo coefficient) at the applied AC field (*V*_AC_) plus the contact stiffness (*k*_c_) augmented by the differential capacitance in the *z*-axis, *V*_AC,_ and the electrostatics at the surface composed of any applied or established DC field (*V*_DC_) and the contact potential voltage *V*_CPD_.^17,24^ This equation relates the observed tip response to the piezoelectric response of the material combined with response due to tip–sample electrostatic interactions. Naturally, a conventional sweep of *V*_AC_ to determine the piezo response (*e.g.*, Fig. 1A), does not compensate for the electrostatic component – the second term of eqn (1). Recent efforts have attempted to minimize this electrostatic response using high *k*_l_ AFM levers to drive *k*_c_ towards zero. This effectively reduces the electrostatic component but does not eliminate it.^27^ Unfortunately, while using stiff, high *k*_l_, AFM levers lowers the electrostatic component with conventional ceramic-based piezoelectric materials, it is only effective in cases where (1) the electrostatic component is small compared to the effective piezoresponse from the material and (2) the elastic modulus of the surface is much greater than the tip.

In the case of soft materials, such as organic and biomaterials, using stiff, high *k*_l_ levers will likely cause significant deformation of the target material. Since DART-PFM uses contact resonance for signal enhancement, the mismatch between the soft surface and stiff AFM lever leads to small tune amplitudes even under large applied fields and thus poor signal to noise. An apt analogy to this situation would be measuring the response of grass with a hammer – compressing the plant and limiting the observable response. Consequently, as proposed in the introduction, softer, low *k*_l_ levers should minimize surface deformation in soft organic and biomaterials; however, they bring additional complications in the form of significant electrostatic contributions to the observed *d*_eff_. Unlike in traditional AC sweep methods here the electrostatic component is expected to be non-zero at zero applied field highlighting the effects of electrostatics on the measurement system. To account for this electrostatic effect, we envisioned sweeping the DC field to accurately determine the electrostatic component of the observed response, as well as the *V*_DC_ point at which the electrostatic response is minimized (Fig. 1B). If successfully realized, we hypothesized this new technique would allow for increased quantitative accuracy in determining the *d*_eff_ piezo response even in soft materials.

**Scheme 1:**
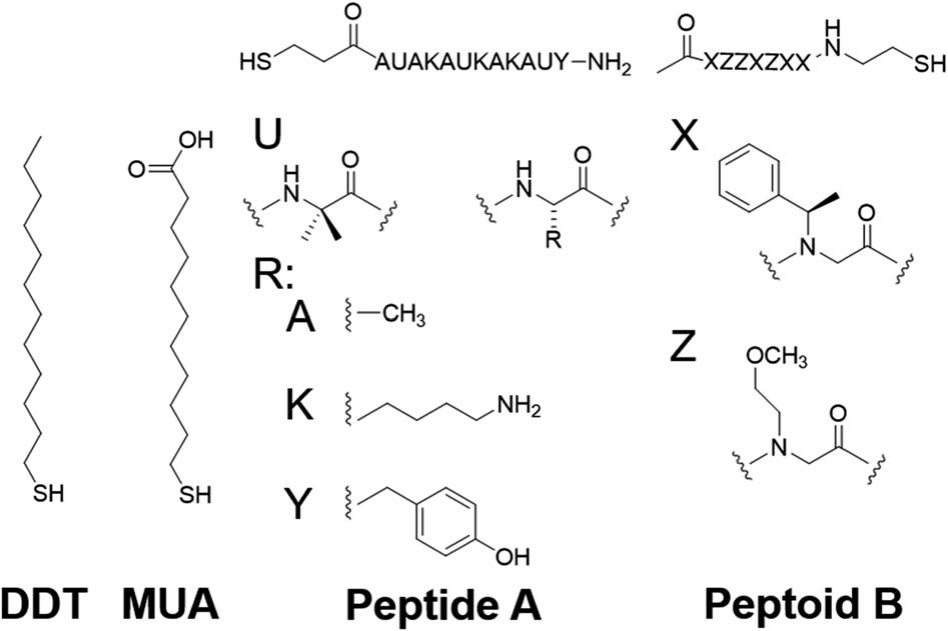
Compounds under study.^16^

**Fig. 2:**
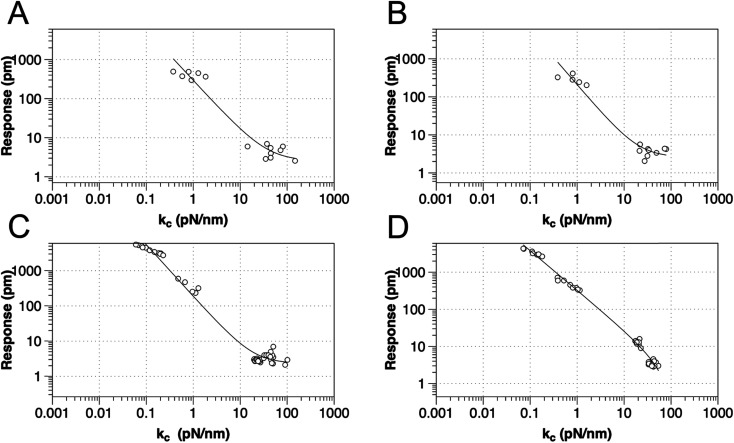
Relationship between tip response (*k*_l_ and *k*_c_ as calculated using eqn (2)) for various SAMs using AFM levers with spring constant from 0.02–2.8 N m^−1^, for (a) DDT, (b) MUA, (c) peptide A and (d) peptoid B respectively. The best-fit line is to *y* = *a* + *bx*^*c*^.

To test the proposed DC field sweeping DART-PFM technique, five different levers were chosen with spring constants (*k*_l_) varying from 0.02 to 2.8 N m^−1^ and used to determine the electromechanical response of four organic self-assembled Au–S monolayers (Scheme 1). These organic SAM systems were chosen due to their innate polar alignment; thus reducing or eliminating any electrostriction or flexoelectric response of the films in conjunction with being non ferroelectric. The SAMs tested included small molecule ligands (DDT, MUA) as well as bio-inspired peptide and peptoid oligomers (A and B) examined in our prior work.^16^ The response of each target film was measured at varying piezo stack voltages, generating varying effective *k*_l_ values. Fig. 2 illustrates the resulting experiment, in which the recorded response for a given target material increases exponentially as the effective *k*_l_ value of the lever used in the measurement decreases. The results confirm that for soft materials like SAMs, using levers with spring constants comparable to the modulus of the material's leads to increased response. In some cases, experimental tip responses reach 250 pm V^−1^, far exceeding previously reported electromechanical response in these soft materials. Though the overall electromechanical response is high, as discussed below, these responses are influenced more by electrostatics than the intrinsic piezo response of the materials (*d*_33_). While the spring constant of the lever (*k*_l_) is shown to influence the response of the films, it is merely contributing to changes in the contact stiffness (*k*_c_).^17,20,24^

As eqn (1) illustrates, while stiffer levers affect the response, it is the contact stiffness (*k*_c_) that directly influences the measurement.^24^ While the distinction may seem subtle, *k*_l_ is merely a single component of the contact. Thus, the spring constant of the contact derives from the lever, the mechanical response of the material in the *x*, *y*, and *z*-axis, influence of surface electrostatics, and any tip–sample meniscus that may be present (*e.g.*, in ambient conditions). Fortunately, these factors can be estimated by applying eqn (2) to the already measured *k*_l_ values (as part of tip–sample tuning in DART-PFM).^17^2
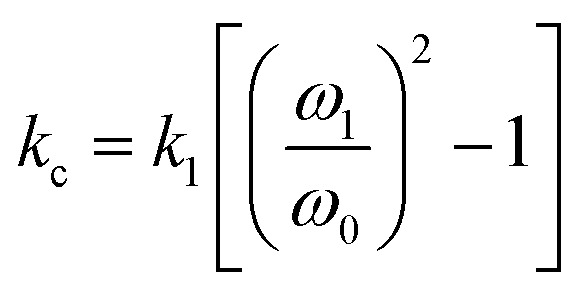


Eqn (2) approximates the spring constant of contact (*k*_c_) from the spring constant of the lever (*k*_l_) by taking the ratio of the resonance frequency of the free lever (*ω*_0_) to the lever in contact with the sample (*ω*_1_) used in the DART experiment.^17^

While the use of stiffer levers is correlated to an increased contact stiffness, using the calculated *k*_c_ values to model tip response, yield better fits (Table 1), reflecting the correct physics due to the higher spring constant of the contact stiffness dominating. The comparable fits of tip response to *k*_l_ values, found in Table S1,† qualitatively maintain the same trend – decreased spring constant yields increased electromechanical tip response, albeit with lower quality of fit (*R*^2^).

**Table 1:** Summary of contact-dependent (*k*_c_) response across four organic self-assembled monolayers, indicating best-fit parameters from the fits in Fig. 2

Material	Constant (*a*)	Coefficient (*b*)	Power (*c*)	*R* ^2^
DDT	2.53	293	−1.30	0.948
MUA	2.56	206	−1.44	0.959
Peptide A	2.32	187	−1.47	0.985
Peptoid B	−2.42	327	−1.07	0.993

However, as indicated in eqn (1), an ideal dependence would yield an exponent of *k*_c_^−1^ (example plot in ESI†), but the values determined by fits in Fig. 2 and Table 1 deviate significantly. In all the organic monolayers, the tip response falls off faster than predicted (*i.e.*, exponents closer to *ca.* −1.3) with increasing contact stiffness. We speculate that this derives from the stiffer levers distorting the monolayers instead of remaining at the interface. The only exception is for the peptoid B SAMs, in which the tip-response curve yields an exponent close to −1.0, suggesting that the peptoid film is significantly stiffer than the other films, as confirmed by AMFM measurements discussed below, and consistent with expectations of a peptoid PPI helix.^27^

While eqn (2) allows an approximate conversion of *k*_l_ to *k*_c_ values, assuming a uniform shift from the fundamental frequency of the lever to the measured frequency of the lever while interacting with surface, *k*_c_ was also measured directly using amplitude modulated force microscopy (AMFM).^10,30–32^ Due to the trends observed in the original *k*_l_ measurements, the *k*_c_ was not directly measured by AMFM for all levers. Only the ASYELEC.01 R2 and the TR400PB (S) levers, 2.8 and 0.09 N m^−1^ respectively, were chosen as the relative extremes of contact stiffnesses observed in the initial study, (Table in ESI†). We note that the measured *k*_c_ values deviate substantially from eqn (2) for stiffer levers, again suggesting that the stiffer levers are distorting the monolayers, effectively limiting the ability of the soft materials to mechanically respond to the applied electric fields.

As mentioned above, while soft levers give higher tip response, they also suffer from greater levels of electrostatic interference than stiffer levers. One way to account for this effect would be to apply a *V*_DC_ to the tip that is equal to *V*_CPD_, thus eliminating the electrostatic term in eqn (1). Intuitively one simple solution would be to measure the *V*_CPD_ by SKPFM, and then apply that *V*_DC_, as has been previously implemented.^27^ The problem arises from the nature of the DART measurement where a *V*_AC_ is applied on top of the *V*_DC_, altering the electrostatic environment around the contact, modulating the intrinsic *V*_CPD_ of the sample. Instead, we swept the DC field to find the point of minimal tip response at which the contact potential equals the applied DC field under a constant *V*_AC_ (Fig. 1B).

**Fig. 3:**
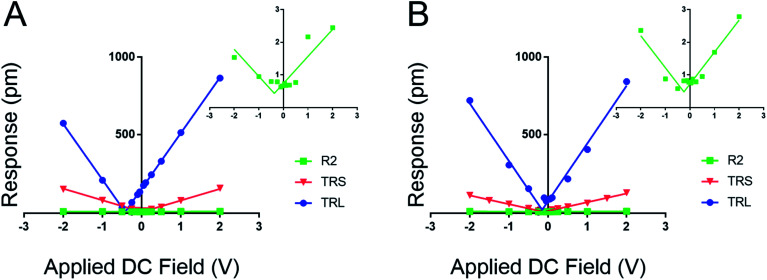
Influence of cantilever contact stiffness on measured film response as a function of DC field. Inset plots highlight the noise floor of the tip response (∼1 pm) when the *k*_l_ is far greater than the modulus of the material. (A) Response of the DDT to varied DC field with three levers ASYELEC.01 R2 (R2, 2.8 N m^−1^), TR400PBS (TRS, 0.09 N m^−1^), and TR400PBL (TRL, 0.02 N m^−1^). (B) Response of peptide A to varied DC field with three levers ASYELEC.01 R2 (R2), TR400PBS (TRS), and TR400PBL (TRL).

The tip response (*R*) is the measured output of the DART experiment after the simple harmonic oscillator (SHO) calculation corrects for the tip–sample resonance enhancement. This tip response can be separated, using eqn (1), into the intrinsic piezoresponse of the material and the electrostatic response. When *V*_DC_ is equal to *V*_CPD_, the electrostatic component of the measurement will go to zero leaving only the mechanical response of the material under the applied field. The organic SAM films are intrinsically polar, permanent piezoelectric materials, since one end is attached *via* an Au–S bond. Consequently, one expects no ferroelectric hysteresis from sweeping *V*_DC_, only two intersecting lines of equal slope proportional to *k*_c_^−1^ d*C*/d*z* (Fig. 1B). The intersection point will represent the piezoelectric response *d*_eff_ × *V*_AC_. The results are highlighted in Fig. 3 and summarized in Table 2, in which three different AFM levers are used on two different organic SAMs.

**Table 2:** Coefficient values and calculated *d*_eff_ from tip response as a function of applied DC voltage at constant *V*_AC_ of 4.0 V

Material	*k*_l_ (N m^−1^)	*V*_CPD_ (V)	Slope (A)	*R* ^2^	*d*_eff_ (pm V^−1^)
DDT	2.8	−0.369	0.827	0.818	0.12
DDT	0.09	0.001	75.1	0.998	−0.28
DDT	0.02	−0.419	360	0.999	−0.32
Peptide A	2.8	−0.245	0.979	0.921	0.12
Peptide A	0.09	−0.111	56.3	0.995	−0.16
Peptide A	0.02	−0.153	373	0.987	3.2

Fig. 3 establishes that the proposed new method works for fixed polar molecular monolayer films. The technique is demonstrated on two SAMs: one piezo active peptide^16^ and a control of DDT, used to highlight the natural polarization of organic SAMs when adsorbed to a metallic surface. The high electrostatic component of the low *k*_l_ levers is easily compensated through the new method. The results point to a piezoresponse range of −0.33 to 0.11 pm V^−1^ for DDT and −0.16 to 3.2 pm V^−1^ for peptide A. The measured *d*_eff_ of peptide A using the 0.02 N m^−1^ lever is significantly larger than the values determined with the stiffer levers.

Further, by highlighting three different spring constant levers ranging from 0.02 to 2.8 N m^−1^ the results from Fig. 2 can be reaffirmed. Here, film response increases with decreasing *k*_l_ due to electrostatic effects, reducing the maximal response at 2.0 *V*_DC_ and 4.0 *V*_AC_ from near 1000 pm to ∼3 pm. These results represent a greater than 300-fold decrease in measured response; moreover the inset charts in Fig. 3 demonstrate that at high *k*_c_, relative to the sample material, the instrument sensitivity bottoms out, effectively identifying the noise floor of the measurement technique. The inset charts emphasize the trend towards higher *R*^2^ values where at high *k*_c_ and *k*_l_, response is sporadic and hard to model in contrast to the low *k*_l_ levers. The increase in sensitivity is further confirmed by the changes in the tune amplitude, at the described set points, from <2 V to >50 V. These, results reflect the benefits of the new method by demonstrating increased precision in the determination of the *d*_eff_ for soft monolayers through enhanced signal to noise ratios.

Based on the evidence in Fig. 2 and 3, the use of soft, low-spring-constant the TRS levers (0.09 N m^−1^) are less likely to perturb organic monolayers, and the DC-sweep DART-PFM technique enables separation of inherent piezoelectric response of a material from the electrostatic components to tip response. Consequently, TRS levers were used with DC-sweep DART-PFM across four organic SAMs and a quartz crystal microbalance (QCM). The latter serves as a non-ferroelectric control with known piezoresponse (*d*_33_), while DDT and MUA SAMs were used as control organic monolayers with low expected piezoresponse, but varying hydrophobicity. If the contact stiffness depends on the effects of a meniscus at the tip sample interface under ambient conditions, modulating from a hydrophobic DDT monolayer to a hydrophilic MUA monolayer should reveal such effects on measured electromechanical response. Peptide A and peptoid B represent helical piezoactive materials with different backbone motifs that give rise to differences in helical propensity.^16^

**Fig. 4:**
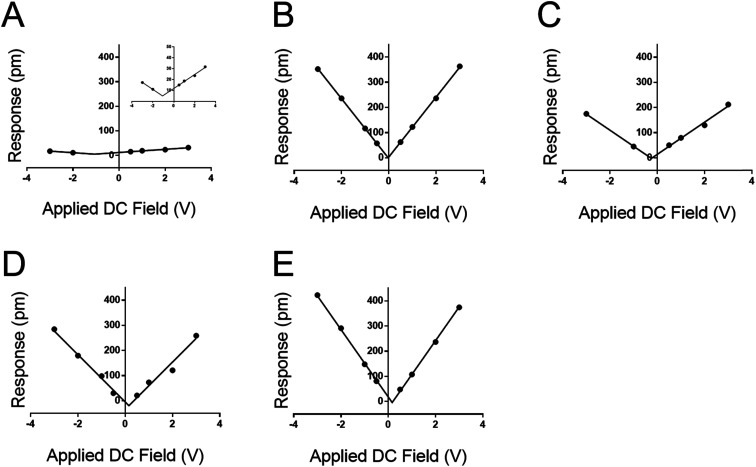
PFM tip response from sweeping the DC field of several materials using TRS levers, and an applied AC field of 3.0 V. Response of (A) quartz crystal microbalance surface; inset provides rescaled *y*-axis, (B) DDT, (C) MUA, (D) peptide A, and (E) peptoid B. Note that all four organic monolayers show profoundly greater DC-field (electrostatic) response than the QCM surface as reflected in the slope of the DC-dependent response.

The DC-sweep DART-PFM response of these films under a constant 3.0 *V*_AC_ field is illustrated in Fig. 4 and compiled in Table 3. The resulting field plots yield *d*_eff_ of the varying materials. QCM stands out with a *d*_eff_ value consistent with literature (*i.e.* 1.68 pm V^−1^*vs.* 2.3 pm V^−1^),^19^ but the observed tip response (*e.g.*, Fig. 4a) is much smaller, compared to the other monolayer samples. The low slope indicates that the magnitude of the *d*_eff_ in quartz is not significantly different from that of the monolayers, but its electrostatic component is minimal compared to the monolayer films. This likely indicates that the ability of quartz to build a large differential capacitance in the *z*-axis is significantly smaller in comparison to the SAMs. Further, these results reconfirm previously reported conclusions that the helix forming peptide and peptoid have higher piezo electric coefficients than DDT and MUA.^16^

**Table 3:** Coefficient values and calculated *d*_eff_ from tip response as a function of applied DC voltage at constant *V*_AC_ of 3.0 V using 0.09 N m^−1^*k*_l_^−1^ levers

Material	*V*_CPD_ (V)	Slope (A)	*R* ^2^	*d*_eff_ (pm V^−1^)
QCM	−1.06	6.40	0.993	1.68
DDT	−0.022	119	0.999	0.100
MUA	−0.249	63.9	0.991	−0.560
Peptide A	0.157	94.1	0.975	−6.42
Peptoid B	0.165	134	0.999	−1.35

More significant than the magnitude of the tabulated piezo coefficients in Table 3 is the sign. Noticeably three out of four SAMs have a negative *d*_eff_, indicating that they compress under an applied field. Only DDT produced a positive *d*_eff_, albeit close to zero. This negative piezo response differs from conventional piezo ceramics such as ZnO or PZT, but is similar to that observed in PVDF and a variety of piezoelectric materials.^33–35^ Thus, the new method not only determines positive, but also negative piezoresponse, even at low applied voltages.

**Fig. 5:**
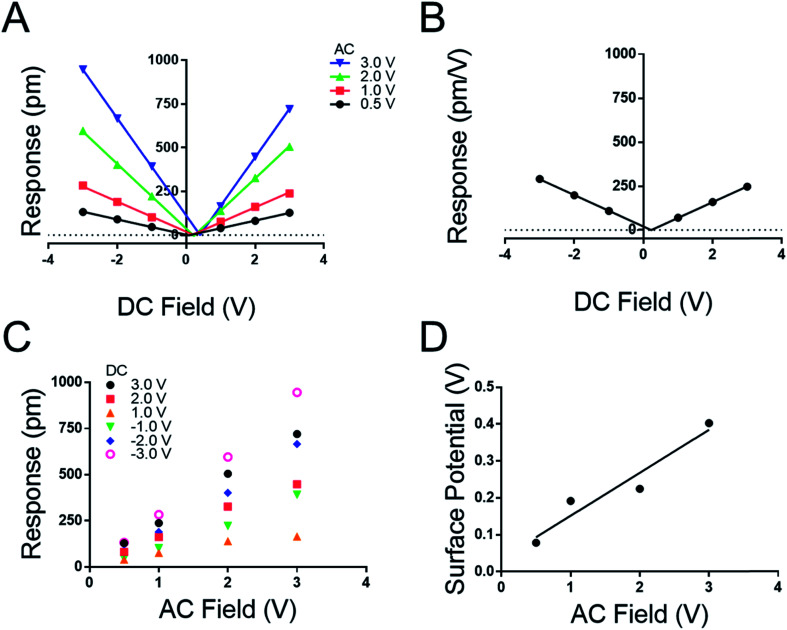
(A) Effect of applied DC field on peptoid B film response at various AC fields using 0.09 N m^−1^ levers. (B) DC-dependent response. (C) PFM response as a function of *V*_AC_ with specified constant DC fields. (D) Measured surface potential as a function of applied *V*_AC_.

We note both the *d*_eff_ and *V*_CPD_ from the peptide A monolayer shifts by applying different *V*_AC_ between Fig. 3 and 4 (4.0 V_AC_ and 3.0 V_AC_ respectively). To test if the *V*_CPD_ and *d*_eff_ is subject to shifting under various experimental conditions a film of peptoid B was tested against four different AC voltages sweeping through six DC voltages at each AC voltage. Extracting the surface potential under experimental conditions from Fig. 5A and comparing it to the applied AC field a linear trend emerges. As the applied electric field increases under the experimental conditions so does the *V*_CPD_. This indicates that a static *V*_DC_ determined by sKPFM cannot be used directly to eliminate the electrostatic component of the measured response, as has been previously suggested.^27^ Further Fig. 5A represents the equivalent of eight experimental runs on one sample using the more traditional AC sweep method, thus confirming the repeatability of the new measurement system and the lack of dielectric brake down of the films due to the applied fields.

Fig. 5A highlights that the maximal response of the film increases with increasing *V*_AC_ as suggested in conventional piezoelectric materials and measurement techniques. To confirm this, a map was extracted from Fig. 5A to generate 5C where the response of the film is plotted against the applied *V*_AC_ at each DC voltage, recreating the conventional approach to the determination of *d*_eff_ by DART-PFM. This exercise emphasizes the ability of the new DC sweep method to remove the effects of electrostatic response and thereby reduce the variation in the measured *d*_eff_. The new method gives the *d*_eff_ of peptoid B to be −0.24 ± 1.36 pm V^−1^ in comparison to the traditional method with the electrostatics unaccounted for at 188.50 ± 39.25 pm V^−1^. To supplement these conclusions the points at each *V*_DC_ in Fig. 5A were averaged with *V*_AC_ removed to generate Fig. 5B. In Fig. 5B the variation at each point is so insignificant that the error does not show on the plot. As expected, the calculated *d*_eff_ from Fig. 5B agrees with Fig. 5A at −0.223 pm V^−1^. Though, these figures are not identical to the *d*_eff_ values determined in Fig. 4, they are within error of each other (Table 4).

**Table 4:** Coefficient values and calculated *d*_eff_ from tip response as a function of applied DC voltage on peptoid B at constant *k*_l_ of 0.09 N m^−1^

*V*_AC_ (V)	*V*_CPD_ (V)	Slope (A)	*R* ^2^	*d*_eff_ (pm V^−1^)
3.0	0.403	277.6	0.999	0.360
2.0	0.225	184.9	0.999	−2.18
1.0	0.192	85.99	0.996	3.37
0.5	0.079	43.86	0.999	−2.51
NA	0.227	89.67	0.999	−0.223

The comparison between the molecular monolayers and quartz highlights a significant shift of material response to an applied field. The slopes of the plots in Fig. 3 represent the electrostatic component of the material response. When comparing the materials there is a significant shift in the slope of the fits indicating a variation in the effect of electrostatics on the reported response. Quartz has a fundamentally shallower slope than any of the molecular films. Likely the applied AC field or the differential capacitance in the *z*-axis are the influencing factors. The AC field however is uniformly applied at 3.0 V across all samples and accounted for when the final response is computed. In addition, a humidity-controlled chamber held at approximately 20% provides no likely outside source for field augmentation, ensuring little to no variation in the meniscus formed at the tip–sample interface. Hence, the contribution from the differential capacitance in the *z*-axis is likely the source of the discrepancy in the overall measured response. This difference in capacitance is likely due to a difference between the relatively high dielectric constant of quartz (*ε* ∼ 4) compared to the lower dielectric constant of the SAMs (MUA*ε* ∼ 2).^36,37^

To test this hypothesis, several conventional hard-ceramic piezoelectric materials were tested, in addition to the non-ferroelectric quartz material sampled above, including: ferroelectric PZT (∼1 cm thick), PPLN (∼1 mm thick), and a second non-ferroelectric material ZnO (∼1 mm thick) (results in ESI†). In all three cases, the technique also worked for minimizing the electrostatic effect on tip response. Only ZnO gave responses indicating a large electrostatic effect from the measured response (see ESI†). The testing of PZT and PPLN mirrored the results of quartz, where the slopes of the fits are shallow, but present higher baseline piezo response. These results confirm that soft-molecule based piezoelectric materials are fundamentally different from classical ceramic based materials and must be analyzed with new methods that allow for operation at higher signal to noise ratios while simultaneously removing the electrostatic component of the response. This has been demonstrated to be achievable by alternatively sweeping the *V*_DC_ instead of the *V*_AC_ and finding the point of inflection where the *V*_DC_ is equal to the *V*_CPD_ and extracting the *d*_eff_ from that point instead of the slope of the fit.

## Conclusions

This work has coupled multiple AFM techniques together to establish and validate a new method for quantitatively separating the electrostatic component from the purely piezoelectric response of low Young's modulus piezo-active materials using DART-PFM. We find that organic monolayers, and other soft electromechanical materials, require the use of low spring constant tips to better match the elastic modulus of the materials. In turn, this increases the electrostatic component of the tip response, which can be minimized by sweeping the DC voltage until the minimum response is found. In principle, this point should reflect the contact potential of the film. We find through scanning Kelvin probe microscopy that the potentials are close, but effects of applied fields during the DART-PFM experiment modulate the *V*_DC_ potential that minimizes the electrostatic tip response. Elastic AMFM results established the necessity to match lever stiffness (*k*_l_) with the modulus of the material under study. Simultaneously, AMFM results confirmed that the contact stiffness (*k*_c_) is directly influenced by the *k*_l_, yet *k*_c_ is the optimal parameter for the accurate determination of the piezoelectric coefficient, unlike previous reports.^27^

We find incredibly large electromechanical tip responses, nearing 250 pm V^−1^, which derive from large differential capacitance of the films rather than the innate piezoresponse. This large electrostatic component from organic monolayers is in stark contrast to a range of inorganic materials studied, which may show greater intrinsic piezoresponse, but much lower electrostatic components. We speculate that while the organic monolayers have lower dielectric constants than piezo ceramics such as PZT, the differential capacitance is high due to their lower elastic modulus and thin layer thickness (*e.g.*, ∼2 nm).

The new method of DC-sweep DART-PFM was used to determine the *d*_eff_ piezoresponse and electrostatic components of four organic monolayers and four conventional inorganic piezo materials. The method finds peptide and peptoid SAMs with both positive and negative piezo response and, coefficients in agreement with previously reported values.^16^ Control molecular SAMs composed of DDT and MUA show close to zero piezoresponse. While scans across multiple films and different AC voltages do affect the measurement somewhat, the DC-sweep DART-PFM technique shows much improved reproducibility relative to previous efforts using varied AC voltages with DART-PFM.

We believe this new technique will improve accurate measurements of electromechanical response in organic and biomaterials. Moreover, the large electrostatic component of electromechanical response found in organic materials can likely be utilized for sensing or other applications.

## Experimental methods

### Monolayer formation

Solvents and reagents were purchased from Sigma-Aldrich without further purification. Biogold substrates were purchased from Thermo Scientific and consist of a glass substrate with a titanium (10 nm) adhesion layer and gold (100 nm). The peptide and peptoid were synthesized and purified following procedures described previously.^16^ Gold-thiol based self-assembled monolayers were prepared from 1.0 mM solutions of dodecane thiol (DDT) or mercaptoundecanoic acid (MUA) in ethanol, peptide in water, and peptoid in acetonitrile. The various solvents were used to ensure maximum solubility of target molecules and have no bearing on SAM formation. Substrates were prepared for SAM formation by multiple ethanol and water washings followed by a 15 minute sonication in the solvent used for deposition (ethanol for MUA/DDT, water for peptide and acetonitrile for peptoid). After the corresponding solvent wash, substrates were rinsed with solvent and dried with N_2_. SAMs were formed by placing clean/dry substrates into 1.0 mM thiol ligand solution for 24 hours in ambient conditions. After the deposition period, samples were removed from solution rinsed, dried with N_2_, covered and placed in a desiccator for a minimum of one hour before analysis. All samples were stored under vacuum conditions in a UV blocking container to prevent thiol oxidation.

### Equipment

All atomic force microscopy (AFM), scanning Kelvin probe force microscopy (sKPFM), piezo force microscopy (PFM), and amplitude modulated force microscopy (AMFM) experiments were carried out on an Asylum Research model MFP-3D SPM. PFM experiments were conducted using dual-AC resonance tracking (DART-PFM) mode. Three sets of cantilevers consisting of six individual probes of varying spring constants were used: ASYELEC-01 R2 (R2), Asylum Research, are iridium-coated conductive silicon probes with a 70.0 ± 19.5 kHz free air resonance frequency, and a ∼280 kHz contact resonance. The R2 has a free air spring constant of 2.8 ± 1.4 N m^−1^. HQ: NSC36/PT (NSC: A, B, and C), MikroMasch, are platinum-coated conductive silicon probes with three independent levers per chip. The levers have a 90 ± 65, 130 ± 98, 65 ± 45 kHz free air resonance frequency for levers A, B and C respectively, giving a ∼340, 520, and 260 kHz contact resonance for each lever. The NSC levers have a free air spring constant of 1.0 ± 2, 2.0 ± 4.5, and 0.6 ± 1.25 N m^−1^. TR400PB (TR: S and L), Asylum Research, are gold-coated conductive silicon nitride probes with a 32.0 ± 14.5 and 10.0 ± 7 kHz free air resonance frequency, but a ∼120 and 40 kHz contact resonance. The TR levers have a free air spring constant of 0.09 ± 0.12 and 0.02 ± 0.02 N m^−1^ respectively.

### DART

DART experiments were conducted at multiple tip–sample AC, and DC biases ranging from |0–4| V. Deflection was set to −0.30 V with a tune *z*-voltage of ∼15 V and a scan *z*-voltage of ∼−7.0 V, to maximize signal and ensure stable contact between probe and sample during scanning, unless otherwise stated. Relative humidity was maintained below 30% with a dry N_2_ purge inside the AFM enclosure. Each sample was examined in a 1.0 μ × 1.0 μm area with a rate of 0.75 Hz at a 90° scan angle to minimize topological artifacts. The topography, piezo-response amplitude and phase images were recorded and q-corrected to account for tip–sample resonance amplification using the built-in simple harmonic oscillator (SHO) function.^16,24^ Histograms of the resulting q-corrected piezo-response amplitude were generated, and the mean value of the distribution was extracted and correlated with the appropriate applied DC and AC fields, as discussed below.

### SKPFM

SKPFM measurements were conducted solely with the R2 levers to attain the contact potential difference (*V*_CPD_) of each target material. Deflection was set to ∼0.0 V *via* tuning, with a scan *z*-voltage of 100 V. Start and delta heights were set to 10 nm for all contact potential images (NAP scanning in Asylum software) with a trigger voltage of 800 mV. A static 1.0 V DC field was established for each measurement with no sample grounding due to the dielectric nature of the monolayers being examined. The implemented scan rate was 0.5 Hz at a 90° scan angle.

### AMFM

AMFM measurements were conducted with R2 and TRS levers to represent the contact stiffness across the range of the cantilever *k* values represented. Mirroring conditions used in DART scans a deflection of −0.30 V with tune/scan *z* voltages of ∼15.0 V and −7.00 V respectively were used. The manufacture provided tip radius for TRs = 42 nm and 25 nm for R2 were used to model tip–sample interactions assuming spherical contact. Scan areas of 10.0 μm × 1.0 μm.

## Conflicts of interest

The authors declare no competing financial interests.

## Supplementary Material

NA-001-C9NA00638A-s001
