# Supplementary material for: Accurate electromechanical characterization of soft molecular monolayers using piezo force microscopy
Source: Nanoscale Adv. 2019 Nov 1;1(12):4834–43. doi: 10.1039/c9na00638a (PMC9416907; doi:10.1039/c9na00638a)
Supplement: NA-001-C9NA00638A-s001 [file NA-001-C9NA00638A-s001.pdf]

Electronic Supplementary Material (ESI) for Nanoscale Advances.
This journal is © The Royal Society of Chemistry 2019

Accurate Electromechanical Characterization of Soft Molecular Monolayers using Piezo Force Microscopy

Nathaniel C. Miller, Haley M. Grimm, W. Seth Horne, Geoffrey R. Hutchison

Department of Chemistry, University of Pittsburgh, Pennsylvania 15260, United States

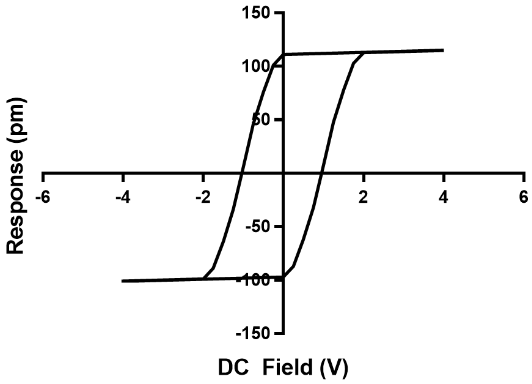

Figure 1: Suggested V_{DC} sweep technique on non-fixed polar piezoelectric materials where the remnant polarization switches under the coercive field.

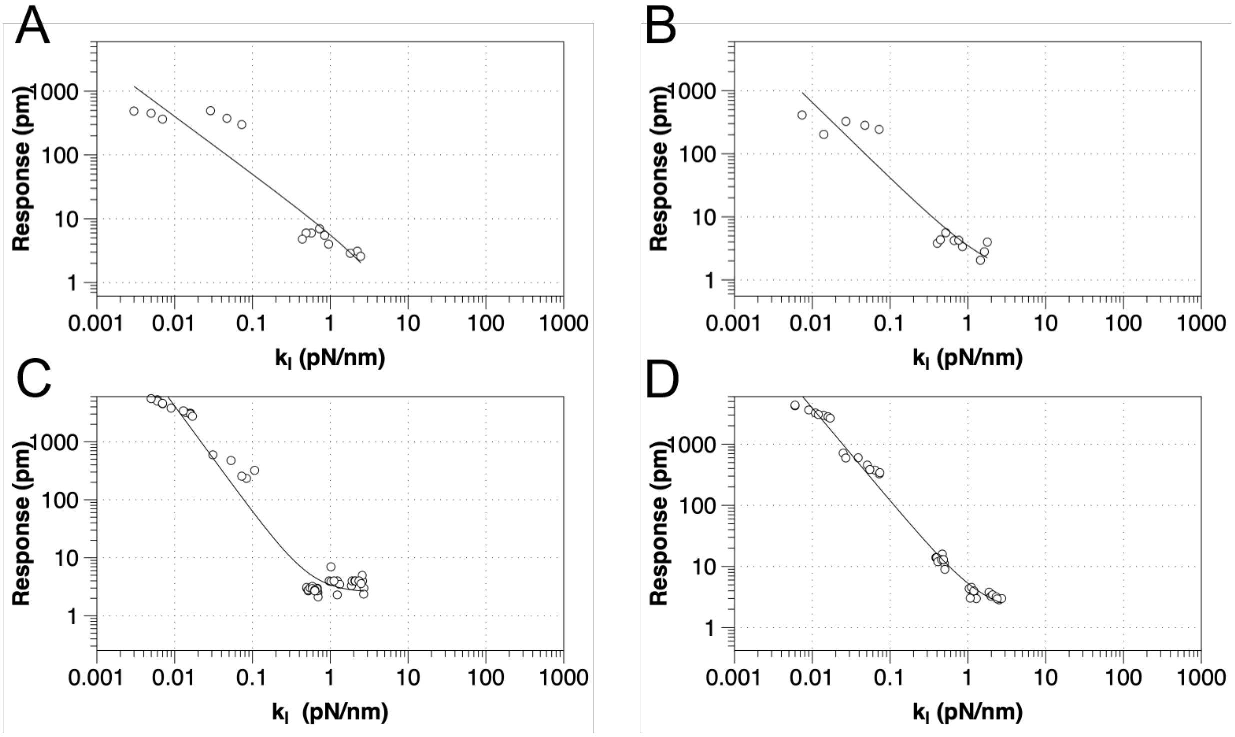

Figure 2: Relationship between tip response and k_l for various SAMs using AFM levers with spring constants from 0.02-2.8 N/m, for (a) **DDT**, (b) **MUA**, (c) peptide **A** and (d) peptoid **B** respectively. The best-fit line is to $y = a + bx^c$.

Table 1: Summary of tip-dependent (k_l) response across four organic self-assembled monolayers, indicating best-fit parameters of tip response to $a + bx^c$.

Material	Constant (a)	Coefficient (b)	Power (c)	R^2
**DDT**	-0.786	6.35	-0.901	0.886
**MUA**	0.973	2.52	-1.21	0.903
**Peptide A**	2.51	0.899	-1.83	0.965
**Peptoid B**	1.73	3.57	-1.52	0.988

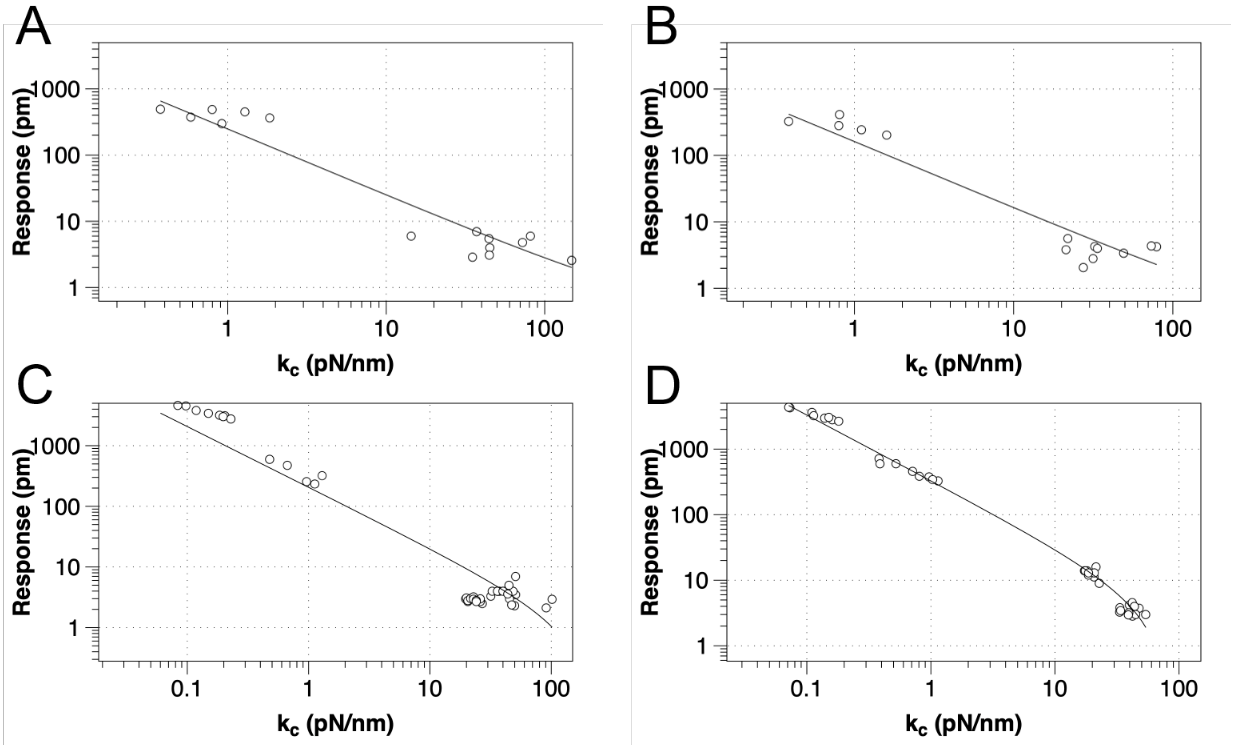

Figure 3: Relationship between tip response and k_c for various SAMs using AFM levers with spring constants from 0.02-2.8 N/m, for (a) **DDT**, (b) **MUA**, (c) peptide **A** and (d) peptoid **B** respectively. The best-fit line is to $y = a + bx^{-1}$.

Table 2: Summary of tip-dependent (k_c) response across four organic self-assembled monolayers, indicating best-fit parameters of tip response to $a + bx^{-1}$.

Material	Constant (a)	Coefficient (b)	R^2
**DDT**	0.342	247	0.932
**MUA**	0.239	162	0.927
**Peptide A**	-1.02	207	0.944
**Peptoid B**	-4.24	331	0.992

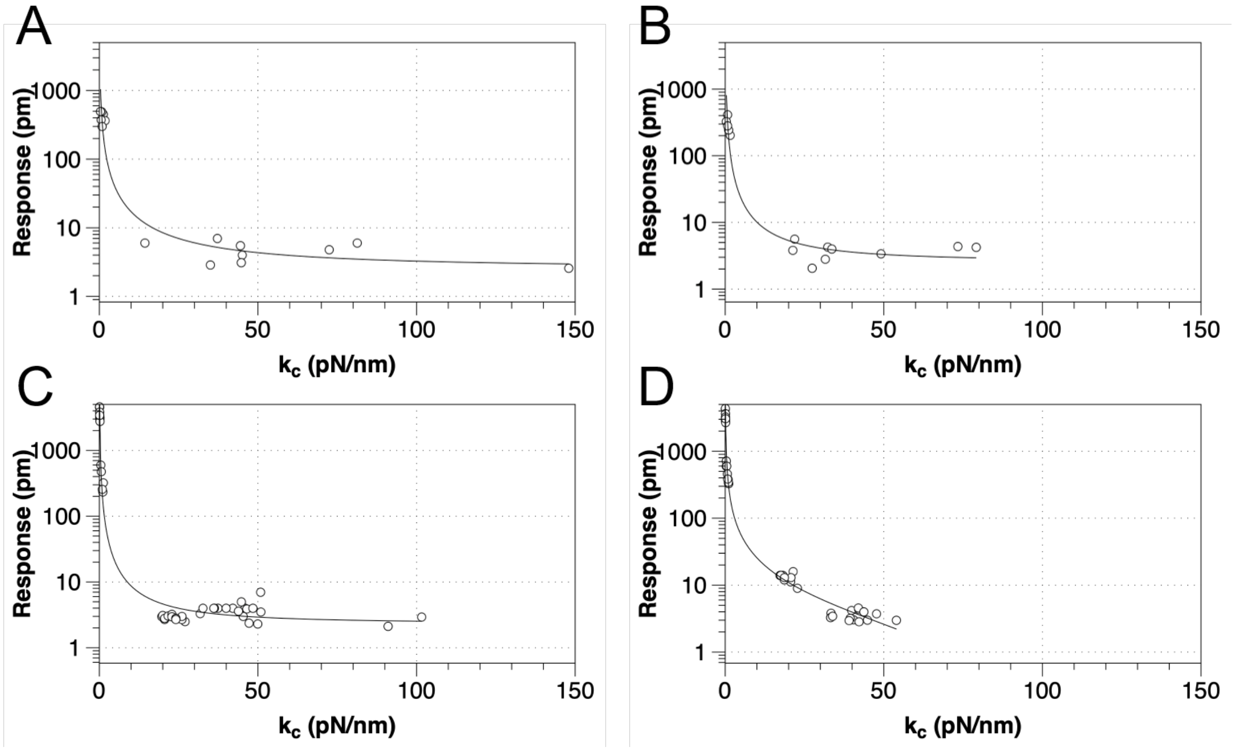

Figure 4: Relationship between tip response and k_c for various SAMs using AFM levers with spring constants from 0.02-2.8 N/m, for (a) **DDT**, (b) **MUA**, (c) peptide **A** and (d) peptoid **B** respectively. The best-fit line is to $y = a + bx^c$. A replotting of Figure 2 from the main text but in log linear scaling to emphasize the asymptotic nature of the fits.

Table 3: AMFM results for the measurement of k_c with corresponding k_l based on implemented lever.

Material	Lever k_l (N/m)	k_l (μN/m)	k^* (μN/m)	k^* Error (μN/m)
**QCM**	2.8	1.66×10^6	1684	165
	0.09	4.41×10^4	132.9	54.1
**DDT**	2.8	1.69×10^6	1937	162
	0.09	3.69×10^4	131.3	35.0
**MUA**	2.8	1.66x10e^6	1272	197
	0.09	3.51×10^4	103.5	35.8
**Peptide A**	2.8	1.68×10^6	1174	158
	0.09	3.59×10^4	26.77	8.13
**Peptoid B**	2.8	1.73×10^6	1244	140
	0.09	3.41×10^4	65.10	26.4

Table 4: Coefficient values and calculated d_{33} from tip response as a function of V_{AC} on peptoid B using 0.09 N/m k_l levers at varying V_{DC}.

V_{DC} (V)	R^2	Intercept (pm)	d_{eff} (pm/V)
3.0	0.998	7.19	241
2.0	0.995	13.9	148
1.0	0.956	22.8	50.6
-1.0	0.991	-32.6	137
-2.0	0.996	-35.7	229
-3.0	0.999	-38.0	325

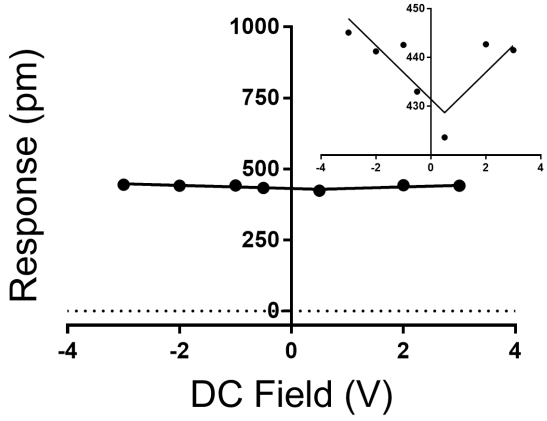

Figure 3: PFM tip response from V_{DC} sweep technique on PZT at 3.0 V_{AC} with R2 levers (2.8 N/m). The resulting slope of the fit was 5.51 pm/V_{DC} with an R^2 value of 0.704. With a calculated d_{eff} of 143 pm/V_{AC}.

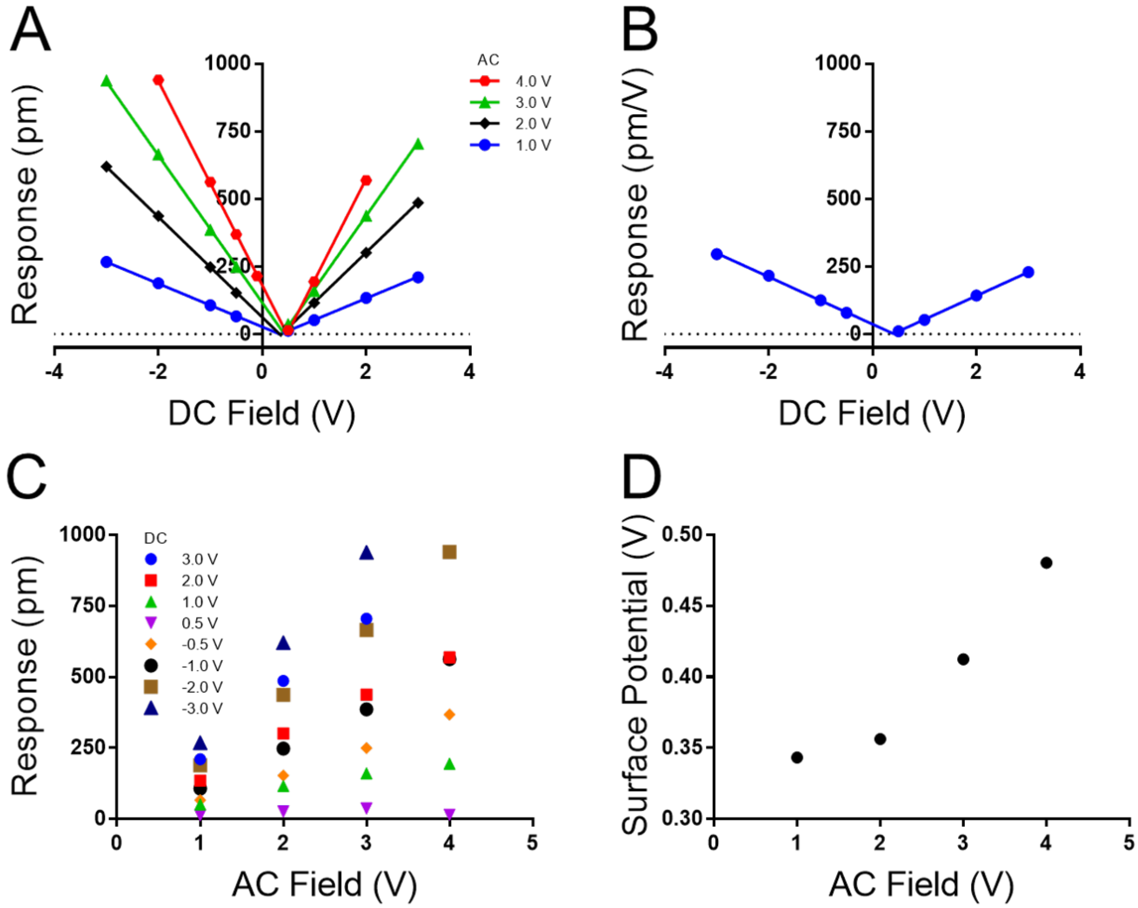

Figure 4: (a) PFM tip response from V_{DC} sweep technique on ZNO with TRS levers (0.09 N/m) at varying V_{AC}. (b) DC- dependent response. (c) PFM response from V_{AC} at indicated V_{DC}. (d) Measure V_{CPD} as a function of V_{AC}.

Table 5: Coefficient values and calculated d_{33} from tip response as a function of V_{DC} on ZNO using 0.09 N/m k_l levers at varying V_{AC}.

V_{AC} (V)	V_{CPD} (V)	Slope (A)	R^2	d_{eff} (pm/V)
4.0	0.480	278	0.999	0.040
3.0	0.412	185	0.999	1.61
2.0	0.356	86.0	0.996	-1.54
1.0	0.343	43.9	0.999	-0.291
NA	0.401	87.5	0.999	1.90

Table 6: Coefficient values and calculated d_{33} from tip response as a function of V_{AC} on ZNO using 0.09 N/m k_l levers at varying V_{DC}.

V_{DC} (V)	R^2	Intercept (pm)	d_{eff} (pm/V)
3.0	0.996	-27.5	247
2.0	0.997	0.675	144
1.0	0.980	12.2	47.3
0.5	0.996	0.740	12.3
-0.5	0.995	-41.9	100
-1.0	0.997	-50.3	151
-2.0	0.999	-62.7	248
-3.0	0.999	-62.8	336

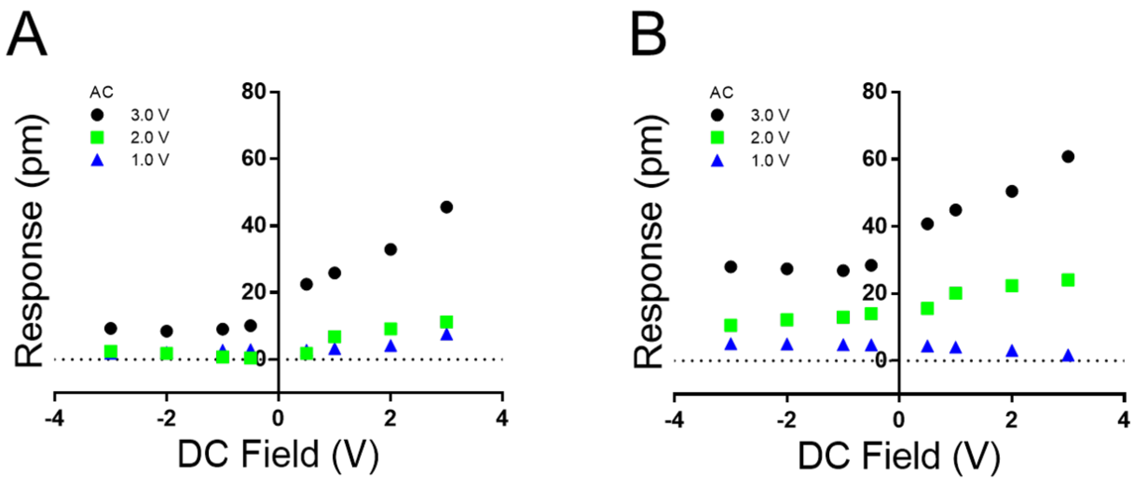

Figure 5: PFM tip response from V_{DC} sweep technique on PPLN at various V_{AC} using R2 levers (2.8 N/m). (a) and (b) represent measured response of PPLN with phase up (+180°) and phase down (-180°) respectively.

Table 7: SKPFM results for the measurement of V_{CPD} for various materials using 2.8 N/m levers.

Material	V_{CPD} (mV)	Error (mV)
DDT	172	5.95
MUA	-198	19.6
Peptide A	-139	16.2
Peptoid B	-362	23.6
QCM	1100	161
ZnO	-745	160
